# Cancer incidence in the south Asian population of California, 1988–2000

**DOI:** 10.1186/1477-3163-4-21

**Published:** 2005-11-10

**Authors:** Ratnali V Jain, Paul K Mills, Arti Parikh-Patel

**Affiliations:** 1 California Cancer Registry, Public Health Institute, Fresno, California, USA; 2 University of California, San Diego, Moores UCSD Cancer Center, Cancer Prevention and Control, La Jolla, California, USA; 3 University of California, San Francisco, Fresno Medical Education Program, Fresno, California, USA; 4 California Cancer Registry, Public Health Institute, Sacramento, California, USA

## Abstract

**Background:**

Although South Asians (SA) form a large majority of the Asian population of U.S., very little is known about cancer in this immigrant population. SAs comprise people having origins mainly in India, Pakistan, Bangladesh and Sri Lanka. We calculated age-adjusted incidence and time trends of cancer in the SA population of California (state with the largest concentration of SAs) between 1988–2000 and compared these rates to rates in native Asian Indians as well as to those experienced by the Asian/Pacific Islander (API) and White, non-Hispanic population (NHW) population of California.

**Methods:**

Age adjusted incidence rates observed among the SA population of California during the time period 1988–2000 were calculated. To correctly identify the ethnicity of cancer cases, 'Nam Pehchan' (British developed software) was used to identify numerator cases of SA origin from the population-based cancer registry in California (CCR). Denominators were obtained from the U.S. Census Bureau. Incidence rates in SAs were calculated and a time trend analysis was also performed. Comparison data on the API and the NHW population of California were also obtained from CCR and rates from Globocan 2002 were used to determine rates in India.

**Results:**

Between 1988–2000, 5192 cancers were diagnosed in SAs of California.

Compared to rates in native Asian Indians, rates of cancer in SAs in California were higher for all sites except oropharyngeal, oesophageal and cervical cancers. Compared to APIs of California, SA population experienced more cancers of oesophagus, gall bladder, prostate, breast, ovary and uterus, as well as lymphomas, leukemias and multiple myelomas. Compared to NHW population of California, SAs experienced more cancers of the stomach, liver and bile duct, gall bladder, cervix and multiple myelomas. Significantly increasing time trends were observed in colon and breast cancer incidence.

**Conclusion:**

SA population of California experiences unique patterns of cancer incidence most likely associated with acculturation, screening and tobacco habits. There is need for early diagnosis of leading cancers in SA. If necessary steps are not taken to curb the growth of breast, colon and lung cancer, rates in SA will soon approximate those of the NHW population of California.

## Background

The south Asian (SA) population of United States was 1,893,723 in the year 2000 [1], and between 1990 and 2000 this population grew in size by 106%. Persons with origins in India, Pakistan, Bangladesh, and Sri Lanka are classified as SA and they are now the third largest Asian subgroup in the United States, comprising 16% of all U.S. Asians. Approximately 21% of SAs in the U.S. reside in California, the state with the largest concentration of SAs. From 1990 to 2000, the number of SAs living in California increased from 168,457 to 343,731 (104% increase) [2,3]. 90% of SAs are Asian Indian (people with origins in India). In the year 2000 the SA population of California comprised 1.15% of the total population and this proportion is increasing.

There are no published studies on the incidence of cancer among SAs in United States, except for one study which reported breast and colon cancer incidence in Asian Indians and that analysis was based on a very small sample size [4]. Another study by Divan et al. reports the current available literature on this issue and emphasizes the need to conduct more studies on cancer incidence and mortality [5]. The reason for lack of cancer studies in this population may be multiple; including controversy regarding which communities are included under the title 'South Asian', the relatively recent growth of this community in the US, and the belief that SAs are part of a 'model minority' and therefore have better health status than other minority groups. In previous studies all Asians have been grouped into one category, which may mask important differences in incidence and survival among various subgroups.

Most of the cancer studies in SAs residing outside of south Asia have been done in the UK or Canada [6]. Many cancer studies have been conducted in the SA population of UK, mainly because they form the largest ethnic minority of UK. Much attention has been focused on breast and lung cancer epidemiology [7-11]. Studies focusing on multiple cancer sites are few [11,12] although some attention has been given to childhood cancers, mainly because childhood cancers are increasing with time [13-16].

Initial studies suggested that English SA rates for all sites combined were lower than the non-SA rates but higher than Indian subcontinent rates (especially for lung cancer in males, breast cancer in females, and lymphomas in both sexes). But a sub-site analysis revealed that, English South Asian rates were significantly higher than the non-SA rates for Hodgkins disease in males, and oral, esophageal, thyroid, leukemias in females, and cancers of the pharynx, liver and gall bladder in both sexes [12].

Recent studies in UK indicate, that younger SA, particularly children are at increased risk of cancer than the non-SA population and although generally cancer rates have fallen over the last decade, they are increasing among SAs [11].

Studies on cancer in the SA population of Canada pertain primarily to cancer screening, and no studies on cancer incidence have been reported [17,18].

Studies of cancer incidence in immigrant populations can provide valuable insights into etiology and changes towards the pattern of disease seen in the host country may indicate environmental factors in etiology [19]. Therefore, in this analysis we have calculated age adjusted rates for cancer in the SA population of California and compared these rates to native Asian Indians (people living in India) as well as the Asian/Pacific Islander (Asian/PI) and non-Hispanic White (NHW) populations of California in the same time period. We also conducted a time trend analysis to study the patterns of cancer incidence in this population for the period 1988–2000. Where appropriate, we have also compared these rates to those reported in Great Britain.

## Methods

The California Cancer Registry (CCR), a population-based registry, commenced operation in 1988. The methodology of the CCR has been fully described by Morris et al. [20]. The CCR collects information on all cancers except for non-melanoma skin cancers and *in situ *cancers of the uterine cervix. Information on several demographic variables, diagnostic variables (including stage at diagnosis, tumor size, histology and grade of tumor), and first course of treatment are collected for all cases. Cases are routinely coded with regard to anatomic stage of disease using the general summary stage schema for 1988–1993 [20], and SEER extent of disease for 1994–1997 [21]. Race and ethnicity are categorized into four mutually exclusive groups in the CCR database: White, non-Hispanic, Black, non-Hispanic, Hispanic, and Asian/Pacific Islander. Under the last category there are further breakdowns for several Asian ethnic groups, including the category 'Asian Indian/Pakistani', which includes people of SA origin.

Our analysis included cancer cases diagnosed during the period 1988–2000. Incidence rates were calculated for this population for all major sites and several specific cancer types. Due to small numbers for some of the cancer sites, the rates for individual years were grouped into three-year categories to reduce the instability of rates. In addition, an age-adjusted trend analysis of the rates was completed for the period 1988–2000 to determine the Annual Percentage Change (APC) (using the non-weighted least squares approach) along with p-values for APCs.

### Ethnic Classification

The SA group is heterogeneous, not only in national origin, sub-ethnicity (and therefore heritable features), and religion, but also in specific details of pertinent lifestyle including alcohol, tobacco, and various levels of vegetarianism. Secondly, individual hospitals, from where most cancer cases are identified by the CCR, do not have the resources to correctly categorize race/ethnicity. Hence many SA cancer patients may be classified as "Asian, not otherwise specified" by the hospital.

Due to the above situation, a British developed software program called 'Nam Pehchan' [22] (literally means name identification in Hindi) was used in this study in order to address the issue of misclassification of race/ethnicity. This software is a computer program for the identification of names, which originate in the Indian subcontinent and Sri Lanka, which collectively we call here "South Asia". It provides a reasonably accurate way of identifying people belonging to "South Asian" and "Other" ethnic groups. It also identifies the religious and linguistic origins of the names where possible. Both surnames and forenames can be matched against the program's stored lists. Given the possibility that different elements of a name may meet with varying recognition from the lookup table, the final result is not simply "South Asian" or "not South Asian", but rather a numeric code indicating the outcome of the search and match process. Knowing the limitations of this program [23], we used this software program, as well as birthplace and a visual case-by-case review to correctly identify approximately 5,200 cancer cases of SA origin, from the 106, 653 Asian/Pacific Islander cancer database at CCR, 1988–2000. We identified 30% more SA cases as compared to CCR (CCR identified approximately 4000 SA cases in the same time period).

### Calculation of incidence rates

Numerators, comprised of all newly diagnosed cancer cases, were derived by applying the Nam Pehchan software to all cancer cases classified as Asian/Pacific Islander by the CCR, 1988–2000. The numerators were coupled with age, gender and yearly specific denominator data for the SA population in California (population counts) obtained from the U.S. Census Bureau. Detailed population counts and demographic characteristics for SA subgroups for both the 1990 and 2000 decennial census are available from the US Census Bureau [2,24]. Electronic population data by age and sex for all SA subgroups were identified and obtained. Hard copy population data for the California 1990 SA subgroups were also identified and key-entered and are available at the cancer registry. Using these census data sets, interpolation between the two decennial censuses was completed and extrapolation back to the years 1988 and 1989 was completed to create the best estimates of the SA subgroups at risk on an age and sex specific basis. The interpolation and extrapolation was done assuming a linear growth in the SA population subgroups. Finally, the subgroup estimates were combined on an age and sex specific basis for each individual year from 1988–2000 to form one SA population group for each individual year. Using these data age-specific and age-adjusted cancer incidence rates were calculated for the time period 1988–2000. We used the 2000 U.S (5-year groups) population as the standard population.

For purpose of comparison between cancer rates in native Asian Indians (living in India) and SAs in California, we calculated Age Standardized Rates (ASRs), using the world standard for the California SAs and compared them to ASRs in India, obtained from the Globocan 2002 [25]. Globocan is a publication of the International Association of Research for Cancer (IARC), and rates for India are for the time period 1993–1997, and cover eight regional registries in India. We used rates from India as our comparison parameter, as 90% of SAs in the U.S. are of Asian Indian origin. In addition, we calculated incidence rate ratios (IRRs) by taking a ratio of California SA ASRs and Indian ASRs, calculated Confidence Intervals (CIs) and determined the significance [26].

### Grouped analysis

Rates for the period of 1988–2000 were divided into 4 time periods by grouping the years of diagnosis into four categories, namely 1988–1991, 1992–1994, 1995–1997 and 1998–2000. Incidence rates were calculated for each of these time periods. We also compared these rates to the Asian/PIs as well as the NHW population of California for the same time periods.

### Time Trend analysis

We performed a time trend analysis for each of the cancer sites separately for males and for females, using the 'age-adjusted trend analysis' feature of SEER-STAT [27]. For this purpose, we used the annual data versus the categorized grouped data. We calculated the Annual Percentage Change (APC) (identifies the percent change by computing the slope of the best-fitting regression line around the data points-rates for each individual years in this case) and p-values for APCs.

## Results

**Figure 1:**
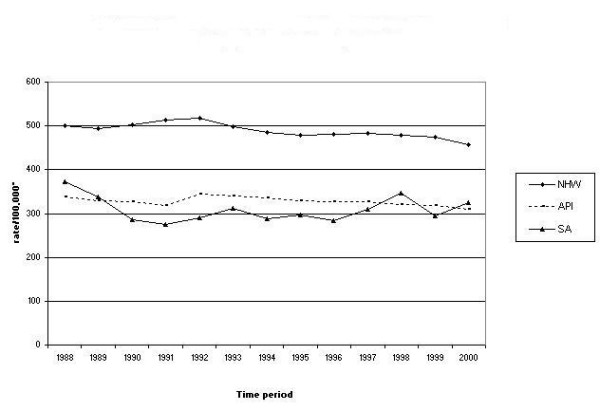
Comparison of invasive cancers, all sites combined, in the SA^1^, NHW^2 ^and API^3 ^population of California, 1988–2000. * Rates are per 100,000 and age-adjusted to the U.S. 2000 standard population (18 age groups). ^1 ^south Asian. ^2 ^White, non-Hispanic. ^3 ^Asian/Pacific Islanders.

In total, 5192 cases of cancer were diagnosed in SA population of California between 1988–2000, including 2411 males, and 2781 females. The median age at diagnosis of cancer was 63 years in males and 54 years in females. A comparison of overall age-adjusted invasive cancer incidence rates for the three ethnic groups revealed that the SA average annual incidence rate was 307.5/100,000, compared to 325.2/100,000 for Asian/PI and 489.1/100,000 for NHW (Figure 1). In the recent years the overall invasive cancer rates for California SAs have been higher than those of the Asian/PIs of the state.

**Figure 2:**
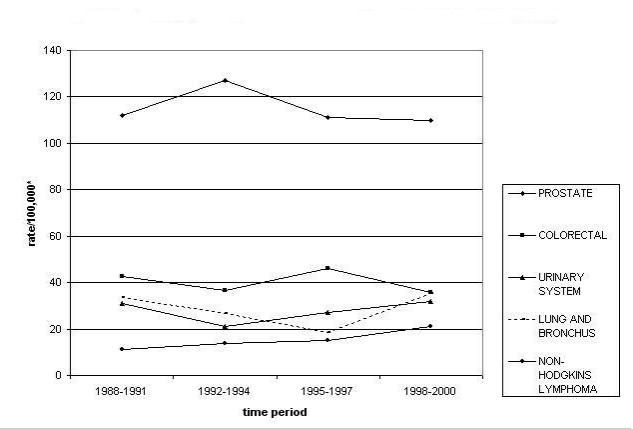
Top five leading cancers with trends in California south Asian males, 1988–2000. * Rates are per 100,000 and age-adjusted to the U.S. 2000 standard population (18 age groups).

**Figure 3:**
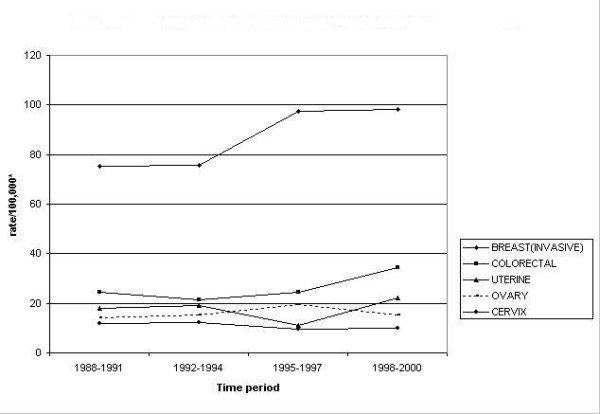
Top five leading cancers with trends in California south Asian females, 1988–2000. * Rates are per 100,000 and age-adjusted to the U.S. 2000 standard population (18 age groups).

Table 1 summarizes the cancer counts by major cancer sites and Figures 2 and 3 show the top five leading cancers and their trends in the SA males and females respectively. Leading cancers in SA males include prostate, colorectal, urinary system, lung and bronchus, and lymphomas. The leading cancer in SA females is breast cancer followed by colorectal, uterine, ovarian and cervical cancer. In this section we have categorized cancers into two groups namely; common cancers (cancers common to males and females) and gender specific cancers (reproductive organ cancers).

**Table 1:** Cancer counts in the south Asian population of California, by cancer sites, 1988–2000.

	**Male and female**	**Male**	**Female**
All Sites	5,192	2,411	2,781
Oral Cavity and Pharynx	160	95	65
Esophagus	52	26	26
Stomach	128	71	57
Colon and Rectum	471	285	186
Liver and Intrahepatic Bile Duct	104	70	34
Gallbladder	48	12	36
Pancreas	83	47	36
Lung and Bronchus	296	188	108
Skin excluding Basal and Squamous	61	38	23
Breast	981	6	975
Cervix Uteri	270	0	270
Corpus and Uterus	145	0	145
Ovary	155	0	155
Prostate	661	661	0
Testis	34	34	0
Urinary System	244	189	55
Brain and Other Nervous System	141	76	65
Thyroid	148	41	107
Hodgkin Lymphoma	56	32	24
Non-Hodgkin Lymphoma	247	143	104
Myeloma	86	49	37
Leukemia	225	138	87

### Comparison of cancer incidence between California SAs and native Asian Indians

Age standardized rates for California SAs and those for India as well as Incidence Rate Ratios (with statistical significance) are presented in Table 2. IRR of more than one indicates that California SAs are at higher risk of developing that particular cancer than the native Asian Indians. Overall, California SA males and females are at double risk for developing cancer than native Asian Indians.

**Table 2:** IRRs and site specific cancer ASRs in India and South Asians in California, 1993–1997.

**SITE**	**SOUTH ASIAN MALES**			**SOUTH ASIAN FEMALES**		
	**India ASR^1^**	**California ASR^2^**	**IRR^3^**	**India ASR**	**California ASR**	**IRR**
**All Sites**	99	199.6	**2.0**	104.4	195.7	**1.9**
Oral Cavity	12.8	5.1	0.4	7.5	4.6	0.6
Esophagus	7.6	2.6	0.3	1.9	2.4	1.3
Stomach	5.7	6	1.1	2.8	5.2	**1.9**
Colon and Rectum	4.7	22.8	**4.9**	3.2	13.4	**4.2**
Liver & Intrahepatic Bile Duct	2.3	5.3	**2.3**	1.1	3.1	**2.8**
Pancreas	1.4	4.5	**3.2**	0.8	4.5	**5.6**
Lung and Bronchus	9	13.9	**1.5**	2	9.9	**5.0**
Prostate	4.6	69.9	**15.2**	~	~	**~**
Breast	~	~	~	19.1	66.6	**3.5**
Cervix Uteri	~	~	~	30.7	8.4	0.3
Corpus Uteri	~	~	~	1.7	10	**5.9**
Ovary	~	~	~	4.9	10.4	**2.1**
Urinary Bladder	3.2	9.4	**2.9**	~	~	**~**
Kidney and Renal Pelvis	1.2	4.9	**4.1**	0.5	2.4	**4.8**
Brain & other CNS	2.6	5.4	**2.1**	1.6	3.6	**2.3**
Thyroid	1	1.5	1.5	1.9	7.6	**4.0**
Non-Hodgkin Lymphoma	3.2	11.2	**3.5**	1.7	10.3	**6.1**
Myeloma	1	4.6	**4.6**	0.6	3.1	**5.2**
Leukemia	3.1	9.9	**3.2**	1.9	6.8	**3.6**

^1^Rates for India obtained from Globocan 2002, rates are an average of 8 regional cancer registries (1993–1997).

^2 ^Age standardised rates (world population), rates are per 100,000 population.

^3 ^Incidence Rate Ratios, ratio of rates in SAs in California vs Indian cancer rates.

IRRs in bold indicate significantly elevated IRRs in California SAs (p < 0.05).

~ indicates cases less than 10 for California SAs, hence rates are not significant enough to report.

#### Common cancers

California SAs were at lower risk of oropharyngeal and esophageal cancers than the native Asian Indian population, which occur very commonly in India. The California SA population was at higher risk for gastrointestinal cancers (namely colorectal, hepatic, and pancreatic cancers). They were also at higher risk for hematopoietic and lymphoreticular and endocrine malignancies. The SA population of California also experienced a higher risk for other organ systems such as, urinary system and brain & CNS cancers.

#### Gender specific cancers

SA men experienced 15 fold risk of prostate cancer than the native Asian Indian population. California SA females experienced higher risk of all reproductive organ cancers except cervical cancer.

### Comparison of incidence rates between SAs and Asian/PIs of the state of California

Incidence rates and time rends between 1988–2000, for California SAs as well as the Asian/PI and NHW population are presented in Table 3 and Table 4.

**Table 3:** Comparison of cancer rates^1 ^in the SA^2^, API^3 ^& NHW^4 ^population of California, 1988–2000.

		**Males**			**Females**		
**Cancer site**	**time period**	**SA**	**API**	**NHW**	**SA**	**API**	**NHW**
**oral cavity and pharynx**	1988–1991	14.6	13.2	20.6	11	7.1	9
	1992–1994	8.3	12.9	19.5	8.2	6.8	8.2
	1995–1997	11.7	13.2	18.9	6.4	6	8.1
	1998–2000	7.7	11.8	17.7	8	6	7.2
							
**esophagus**	1988–1991	7.9	6.3	6.4	2	1.4	2.3
	1992–1994	1.9	4.8	6.8	4.1	1.2	2.1
	1995–1997	5.7	4.9	7.2	3.9	1.1	2.3
	1998–2000	4.1	3.4	7.5	6	1.4	2.3
							
**stomach**	1988–1991	16.6	24.9	12.9	4.9	15.9	5.4
	1992–1994	8.6	24.9	11.4	7.6	15.5	4.6
	1995–1997	11.8	24.2	11	9.8	13.8	4.4
	1998–2000	10.8	20.5	9.7	10	12.2	4.4
							
**liver and intrahepatic bile duct**	1988–1991	10.4	21.6	4.2	5	7.2	1.9
	1992–1994	11	22.6	5	4.3	9.2	2.1
	1995–1997	9.8	24.4	5.6	5.3	9.1	2.3
	1998–2000	10.4	24.5	6.3	6.4	8.5	2.5
							
**gall bladder**	1988–1991	1	1.6	0.8	9.4	3.3	1.2
	1992–1994	1.9	1.6	0.7	9.1	2.4	1.1
	1995–1997	2.4	1.5	0.6	3.2	2.2	1.1
	1998–2000	1.2	1.2	0.6	3.6	1.8	1.1
							
**pancreas**	1988–1991	6.3	12.4	13.3	6.6	8.6	10.2
	1992–1994	4.4	11	12.8	7.3	6.6	9.9
	1995–1997	7.4	9.6	12.5	6.9	7.9	9.4
	1998–2000	10.6	8.9	11.8	3.2	7.5	9.5
							
**lung and bronchus**	1988–1991	33.5	65.3	101.9	19	28.3	59.3
	1992–1994	26.8	64.7	92.6	21.7	26.9	59.5
	1995–1997	18.5	60.8	86.5	18	28.4	60
	1998–2000	35.2	59	79	27	27.3	57.2
							
**colon and rectum**	1988–1991	42.6	57	78.8	24.6	41.9	54.6
	1992–1994	36.8	58.5	71.4	21.5	39.6	49.3
	1995–1997	46.2	58.3	67.7	24.6	39.1	47.9
	1998–2000	35.6	52.4	64.1	34.5	40.1	46.7
							
**brain and other CNS (central nervous system)**	1988–1991	8.2	4	9.4	7.4	3.6	6.5
	1992–1994	10.5	4.7	9.1	5.7	3.7	6.4
	1995–1997	3.7	3.8	8.9	3.9	3.3	6.1
	1998–2000	7.3	4	8.8	8.3	2.6	6.1
							
**urinary bladder**	1988–1991	22.3	15	43.5	5.2	4.4	10.7
	1992–1994	14.8	17	42.3	1.6	4.3	9.9
	1995–1997	18	14.8	41.2	3	4.4	10.1
	1998–2000	18.6	15.1	40.9	3.2	3.7	9.9
							
**kidney and renal pelvis**	1988–1991	8.8	6.6	14.6	2.8	3.8	6.8
	1992–1994	6.2	7	14.2	3.3	3.4	7
	1995–1997	7.9	8.3	14.8	3.2	3.9	7.3
	1998–2000	12.5	8.1	14.8	3.8	3.7	7.2
							
**endocrine (thyroid)**	1988–1991	4	4.2	4.2	7.6	9.7	7.7
	1992–1994	1.3	4.7	3.9	8.7	11.2	8
	1995–1997	2.2	4.6	4.1	9.4	11.5	8.8
	1998–2000	3.2	4.3	4.5	8.5	12.4	10.2
							
**non-Hodgkins lymphoma**	1988–1991	11	15.8	24.4	10.5	10.6	14.6
	1992–1994	13.8	16.7	25.7	16.2	10.6	14.7
	1995–1997	15.2	15.9	25.7	14.7	10.7	15.7
	1998–2000	21.1	15.7	23.3	11	11.4	15.9
							
**Hodgkins lymphoma**	1988–1991	2	1.5	3.6	1.5	0.6	2.8
	1992–1994	2.4	1.5	3.4	1.9	0.8	2.7
	1995–1997	1.7	1.2	3.4	1.5	0.8	2.8
	1998–2000	2.2	1.1	3.3	1.5	1	2.6
							
**leukemias**	1988–1991	10.4	10.9	18	9.6	6.6	10.2
	1992–1994	13.4	10.4	17.5	11.4	7	9.7
	1995–1997	9.6	9.6	16.9	9	6.3	9.8
	1998–2000	16.1	9.2	15.4	8.4	5.8	9
							
**multiple myelomas**	1988–1991	10.5	4.8	6.3	5.1	3.3	4.2
	1992–1994	5.3	4.5	6.4	7.9	2.4	3.9
	1995–1997	9.8	5.2	6.4	2.9	3.2	4.1
	1998–2000	5.6	3.7	5.7	5.5	3	3.6
							
**skin, excluding basal & squamous cell cancer**	1988–1991	8.2	3.9	44.4	2.5	2	20.5
	1992–1994	2.7	4.2	45.8	1.1	1.8	21.8
	1995–1997	4.4	3.9	50	1.9	1.6	28.3
	1998–2000	4	2.8	53.1	2.3	2.5	31.8

^1 ^Rates are per 100,000 and age-adjusted to the U.S. 2000 standard population (5 year age-groups).

^2 ^South Asian.

^3 ^Asian/Pacific Islander.

^4 ^white, non-Hispanic.

**Table 4:** Comparison of rates^1 ^of reproductive organ cancers in the SA^2^, API^3 ^& NHW^4 ^population of California.

**Cancer site**	**time period**	**SA**	**API**	**NHW**
**MALES**				
**prostate**	1988–1991	112.5	78.2	165
	1992–1994	127.8	109.4	197
	1995–1997	111.8	90	153.2
	1998–2000	110.7	87.9	152.2
				
**testicular**	1988–1991	1.5	1.5	6.5
	1992–1994	2.2	1.9	6.5
	1995–1997	0.8	1.6	6.4
	1998–2000	1.9	1.9	7.1
				
**FEMALES**				
**breast (in situ)**	1988–1991	8.1	10.2	19.9
	1992–1994	14.1	12.3	21.7
	1995–1997	13.2	17.1	25.3
	1998–2000	14.6	21.6	29.4
				
**breast (invasive)**	1988–1991	75.2	76	144
	1992–1994	75.5	79	142.3
	1995–1997	97.6	87	146.5
	1998–2000	98.1	91.9	150.8
				
**ovary**	1988–1991	14.3	12.9	19.5
	1992–1994	15.1	13.3	19.3
	1995–1997	19.4	13.4	18.2
	1998–2000	15.1	12.2	18.2
				
**cervix**	1988–1991	11.7	14.7	9.3
	1992–1994	12.1	15.8	8.6
	1995–1997	9.5	13.1	8.4
	1998–2000	10	9.8	7.9
				
**uterus and corpus**	1988–1991	17.8	13.9	28.1
	1992–1994	19	14.8	27.1
	1995–1997	11	14.8	27.1
	1998–2000	22.2	15.9	25.3

^1 ^Rates are per 100,000 and age-adjusted to the U.S. 2000 standard population (5 year age-groups).

^2 ^south Asian.

^3 ^Asian/Pacific Islander.

^4 ^white, non-Hispanic.

#### Common cancers

In general, the SA population of California experienced more brain & CNS cancers, hematopoietic and lymphoreticular cancers than the Asian/PI population of the state. SA females also experienced higher oropharyngeal, esophageal and gall bladder cancer than the Asian/PI women of California. As regards to other cancer sites, the SA population of California was at equal or lower risk than the Asian/PIs of the state.

#### Gender-specific cancers

SA males experienced more prostate cancer than the Asian/PI males and SA females experienced more reproductive organ cancers than the Asian/PI women, except for cervical cancer.

### Comparison of SA rates with NHWs of the state

#### Common cancers

The SA population of California experienced more Gastro Intestinal cancers (mainly hepatic, gall bladder and stomach cancers) and myelomas than the NHW population of the state. SA females experienced more oropharyngeal and esophageal cancers than the NHW women. SA males experienced recent increase in leukemia incidence as compared to the NHW males.

#### Gender specific cancers

As far as the reproductive cancers are concerned, the SA population was at lower risk of these cancers than the NHW population of the state, except for cervical cancer.

### Trends over time

Overall, SA males experienced a decreasing trend of all cancers combined, over the time period of 1988–2000 (APC = -1, p = 0.2), while SA females experienced an increasing trend (APC = 0.9, p = 0.4). Table 5, shows the APCs and their p-values for each individual cancer in the SA population.

**Table 5:** Table showing annual percentage change along with significance in cancer sites in the SA population of California, 1988–2000.

**SITE**	**MALE**		**FEMALE**	
	APC*	**P-VALUE**	**APC**	**P-VALUE**
**All Sites**	-1.0	0.2	0.9	0.4
***common cancers***				
**Oral Cavity and Pharynx**	-3.0	0.5	-4.0	0.1
**Esophagus**	-1.3	0.9	7.2	0.3
**Stomach**	-1.9	0.5	5.6	0.3
**Liver and Intrahepatic Bile Duct**	2.1	0.7	1.2	0.86
**Gallbladder**	~	~	-9.9	0.1
**Pancreas**	3.5	0.5	-1.2	0.8
**Colon and Rectum**	-1.3	0.3	3.5	0.3
**Colon excluding Rectum**	-3.2	0.1	11.2*	<0.05
**Lung and Bronchus**	-2.9	0.3	0.8	0.8
**Skin excluding Basal & Squamous**	-9.3	0.1	~	~
**Urinary Bladder**	0	1	~	~
**Kidney and Renal Pelvis**	8.1	0.2	~	~
**Brain and other CNS**			-1.4	0.7
**NHL**	6	0.1	0.8	0.9
**multiple myelomas**	-2.8	0.6	0.6	0.9
**leukemias**	2.6	0.5	-0.1	1
**Thyroid**	~	~	-1.1	0.8
***Reproductive organ cancers***				
**Breast (In situ)**	~	~	8.8*	<0.05
**Breast (Inavasive)**	~	~	2.3	0.2
**Cervix Uteri**	~	~	-2.1	0.3
**Corpus and Uterus**	~	~	1	0.8
**Ovary**	~	~	-0.2	0.9
**Prostate**	-0.5	0.6	~	~

* Annual Percentage change, numbers in red indicate significant p-values.

~ Annaul percentage change could not be calculated.

#### Common cancers

The SA population of California experienced a significantly decreasing trend of oropharyngeal cancers. On the other hand they experienced an increasing trend of hepatic and renal cancers. In addition, SA males experienced an increasing trend of hematopoietic & lymphoreticular cancers (NHL, multiple myelomas, leukemias) and brain & other CNS cancers. SA females experienced an increasing trend of gastrointestinal cancers (esophageal colon, hepatic, and stomach), lung and thyroid cancers.

#### Gender-specific cancers

As far as the reproductive organs were concerned, SA females experienced an increasing trend of breast and uterine cancers. All other sites experienced either a decreasing or steady trend over time.

## Discussion

The present study reveals several unique cancer patterns among SAs in California. Firstly, the median age at diagnosis of cancer in this population is 58 years compared to 68 years for all other races [28]. Secondly, the most common cancers in the Indian subcontinent are not the most common cancers in SAs of California. The most common cancers among men in India are oral cavity and pharynx, lung, esophagus, laryngeal and stomach cancers [25]. In India, cervical cancer is most common in women, followed by breast, oral cavity, esophagus and ovarian cancer [25]. In India about half the cases among men and one-fifth cases among women are in cancer sites affected by tobacco use (tobacco smoking as well as tobacco chewing) [29], which was not seen in SAs of California.

### Common cancers (cancers common to both males and females)

#### Oropharyngeal cancers

Our findings indicate that California SAs are at lower risk of oral and esophageal cancers than the native Asian Indians. This directly reflects the general tendency of the SA immigrants to avoid use of tobacco products (especially chewing 'paan' (tobacco rolled up in betel nut leaves) and smoking 'bidi' (cigarette made out of tobacco leaves, with no filters) in a foreign country. Besides, majority of SA immigrants in California tend to be educated and do not have such habits even in South Asia.

#### Esophagus cancer

Esophageal cancer is increasing in SA females and is higher than both NHW and Asian/PI females. Such findings of increasing trend are not seen in the SA males. This finding also seems contradictory to the general decreasing trend of oropharyngeal cancers, as esophageal and oropharyngeal cancers share similar etiologies. The etiology of esophageal cancer is mainly associated with consumption of tobacco (smoking or smokeless) and alcohol. In addition Barrett's esophagus, diet and nutrition, reflux disease also play an important role in etiology of esophageal cancer [30,31]. There are no published studies about smoking/tobacco/alcohol use prevalence in the SA population in the U.S. Because of lack of such data we cannot correlate our findings with the smoking prevalence. The rise of esophageal cancer in California SA females as well as histological subtype evaluation of this cancer is needed.

#### Stomach cancer

IRRs suggest that California SA females are at a higher risk for stomach cancer than native Asian Indian females, but this is not true for males. The time trend analysis suggests that male stomach cancer is decreasing, but female stomach cancer is on the rise. Infections with Helicobacter pylori and genetic predisposition of host have been suggested to be the most important causes of stomach cancer in general population [32,33].

#### Cancers of the liver and intrahepatic bile duct

These cancers are of common occurrence in Asians. HBV (hepatitis B virus) infection, with and without aflatoxin exposure, and alcoholic liver cirrhosis are responsible for most cases of hepatocellular cancer in developing countries [34]. There is widespread contamination of foods with aflatoxin and moderately high prevalence of HBV and hepatitis C (HCV) virus-related chronic liver disease in India [35]. IRRs suggest that California SA population is at higher risk (more than two-fold) of hepatic cancers than native Asian Indians. Our findings are similar to the studies done in the past in UK on migrants of Indian ethnicity as well as British ethnicity, to the UK [16,36].

#### Gall bladder cancer

The major causative factors for gall bladder cancer include gallstones and genetic susceptibility, and liver flukes in Asian countries have also been suggested to be causative [37]. In one study done in India, the prevalence of gallstones in adult population was 6.12% (3.07% in males, 9.6% in females) [38]. All these above stated factors could explain our finding of much higher rates in the SA population than Asian/PI or NHW population. Similar findings have been reported by studies in SA immigrants to the UK [12,36,39]. Nevertheless, it is encouraging that there is a significantly decreasing trend of this cancer in California SAs.

#### Colon and rectal cancer

Both SA males and females of California experienced more than four-fold risk of developing this cancer compared to the native Asian Indian population. Studies in the general population estimate that 13% of this cancer can be attributed to being physically inactive, 12% to eating a Western style diet, and 8% to having a first degree relative with colorectal cancer [40]. The diet of Asian Indians in the United States has changed from one featuring low-fat, high-fiber foods to one characterized by higher-fat animal protein, low fiber, and high levels of saturated fat. There is an increased tendency among Asian Indians in America to consume fast foods and convenience foods [41]. The significantly rising trend of colon cancers seen in SA females, which is otherwise a low-risk population, may be related to migration and subsequent acculturation and adoption of Western diet and lifestyle.

#### Lung and bronchus cancers

As compared to the native Asian Indian rates, the SAs of California are at higher risk for this cancer. The five-fold risk in California SA females as compared to the native Asian Indian females and an increasing trend is noteworthy. A decreasing trend of lung cancer in SA males is not in concordance with a recent study done in the UK SA population, which reports recent increase in incidence of lung cancer in both SA men as well as women [7].

#### Non-Hodgkin's Lymphomas (NHL)

IRRs suggest that the California SAs are at a much higher risk (3–6 fold higher risk) of developing NHL than native Asian Indians. In addition, an increasing trend of NHL has been observed in the SA population of California. While the incidence of NHL has doubled in the U.S., etiology of lymphomas remains elusive. Epidemiological studies suggest the role of hereditary factors, immunosuppression, viruses (HIV, EBV, HTLV, H.pylori, HHV8, HCV), chemical and agricultural exposures and other factors in the etiology of NHL [42]. Recent studies have also associated menstrual and reproductive factors (higher parity and early menarche offer a protective effect for NHL) with risk of NHL [43,44]. Lack of immune stimulation/challenge ('hygiene hypothesis') [45] and acculturation could explain the higher risk seen in this population.

#### Leukemias

Three-fold higher risk of developing leukemias in California SAs as compared to the native Asian Indians, and a rising trend of this cancer over time shows similarity with results from UK SA studies [14,16,19]. Types of leukemias and their causes vary widely and are age dependant. Further investigation, especially age specific and leukemia subtype analysis is needed into this finding.

#### Multiple myelomas

IRRs suggest that California SA population experience a much higher risk (four-five fold) of developing myelomas than the native Asian Indians, as well as higher rates than the Asian/PI or NHW population of California. Risk factors for multiple myelomas include, monoclonal gammopathy of unknown significance, chronic immune stimulation (as in infections with tuberculosis, malaria, hepatitis, etc.), autoimmune disorders, and occupational exposures [46]. Every year, approximately two million persons in India develop tuberculosis, and incidence of malaria is 2–3 million cases per year [47,48]. Exposure to these chronic diseases before migration could explain the high rates of myelomas seen in California SAs.

Findings of elevated risk of haematopoietic and lymphoreticular malignancies (lymphomas, leukemias and myelomas) in SAs after migration needs further investigation. Similar results have been reported in SA immigrants of UK [12,16,36].

#### Thyroid cancer

IRRs indicate that California SA females are four times more likely to get thyroid cancer than Indian females; this is not true in males. The incidence of congenital hypothyroidism and prevalence of goiter in India is much higher than the worldwide average [49]. A large fraction of the Indian population suffers from iodine deficiency disorders [50]. The major etiological factors for thyroid cancers have been iodine deficiency and ionizing radiation [51-53]. We cannot explain the higher IRR observed in California SA females.

#### Brain and other nervous system cancers

California SAs experienced higher IRRs of these malignancies as compared to native Asian Indians. SA males experience higher rates of these malignancies than the Asian/PIs and SA females recently experienced higher rates than Asian/PIs as well as NHWs. This finding is not in concordance with the other studies done in the UK SA population [14,16]. These cancers are infrequent in India and frequent amongst the U.S. Whites, making the SA population a low-risk population [54,55]. In spite of being a low-risk population, higher IRRs and rates of these cancers observed in SAs need further investigation.

### Gender-specific cancers

#### Prostate cancer

Prostate cancer is the most common cancer in SA males and has increased from 1988–2000. California SA males experienced fifteen fold-increased risk of this cancer as compared to Indian males. Also, rates are higher in California SA males than in Asian/PIs of California. Epidemiological studies suggest that endogenous risk factors like family history, androgens, race, aging, oxidative stress and exogenous factors including diet and environmental agents have been associated with this cancer [56]. Other studies suggest that screening for this cancer has dramatically increased the number of men with local disease at diagnosis [57]. The fifteen-fold risk of prostate cancer in this population as compared to the native Asian Indians could be explained by early detection (measurement of serum PSA), rather than true differences in underlying risk. The other factors explaining this difference could be lead-time, case identification, detection and reporting biases.

#### Breast cancer

Breast cancer is the number one cancer in the California SA females and they are 3.5 times more likely to develop this cancer as compared to native Asian Indian females. Our time-trend analysis suggests that, although in situ breat cancer diagnosis has significantly increased, invasive breast cancer diagnosis has increased alarmingly more in SA than in Asian/PIs and NHWs. In the general population major risk factors include, late maternal age at first parity (>30 years of age), having one child vs. 4, use of oral contraceptives (OCs), use of hormone replacement therapy (HRT), obesity and alcohol [58-60]. Adoption of above-mentioned lifestyle practices by SA women and inadequate screening could be related to the increase in breast cancer in this population.

#### Cervical Cancer

Although HPV has been proposed as the first identified necessary cause of cervical cancer [61,62], we attribute the decreasing trend and very low IRRs of cervical cancer in California SA women to screening success. California SA women are getting screened at very early stages and hence treated completely as compared to the Indian women (cervical cancer ranks number one in India).

#### Ovarian and uterine cancers

Risk factors for epithelial ovarian cancer include older age, being White, positive family history, nulliparity, infertility, and obesity (high saturated fat and carbohydrate intake), postmenopausal HRT and use of cosmetic talc. Conversely, preventive factors include OC use, vegetable consumption, gravidity, lactation, tubal ligation, and hysterectomy. Genetic influence also plays a role, women with mutations in the BRCA1 or BRCA2 genes having an elevated risk [63-65]. Rates of this cancer in the SA women are higher than the ASIAN/PIs and almost approximating those of NHWs. Almost two-fold elevated risk of ovarian cancer in California SA women compared to native Asian Indian women can be explained by all the adoption of above mentioned western life-style factors.

Similarly uterine/endometrial cancer is a disease of the developed world. Epidemiological studies have shown that majority of the incidence can be attributed to excess body weight (in turn due to 'unopposed estrogens'), lack of physical activity, exogenous hormones and chronic hyperinsulinemia along with genetic predisposition [66,67]. California SA women face a five-fold risk of this cancer as compared to the native Asian Indians and they show much higher rates than the Asian/PIs and their rates seem to be fast approaching the NHWs of the state. Clearly, acculturation can explain these findings.

### Limitations

Certain limitations in the methods employed in this study deserve comment. The assumption of a linear growth of population may not be completely tenable, and various factors such as birth/death rates and immigration/migration related issues could impact patterns of population growth. However, communication with staff at the Los Angeles County Cancer Surveillance program indicated that interpolation performed well when compared to more complex methods of estimation based on year/race/ethnicity/sex and county specific population estimates obtained from the California Department of Finance (state agency in California charged with maintaining intercensal population figures) [personal communication, Dr. Lihua Liu, USC/CSP, December, 2003].

While performing incidence studies on sub-ethnic populations, the issue of small number of cases is inevitable. This could create instability of rates, especially in analyses pertaining to trends over time. To overcome this, forming groups and performing a grouped analysis in those groups was completed.

## Conclusion

Our findings are in general agreement with studies completed in the UK and suggest a strong role for acculturation, screening and lifestyle factors in explaining the patterns of cancer in SA in California. Minor disagreements with findings in UK studies are to be expected, as there are minor underlying differences in methodology. For example, some studies have used absolute numbers for comparison or a proportionate approach for comparison. But most of the studies have reported incidence rates based on data available from the cancer registries and census bureaus/corresponding organizations in UK (with whom we have compared our data).

More studies are needed to evaluate gender differences in this population, especially the rising trend of gastrointestinal cancers seen in SA females vs. males, needs more investigation. Our study also reveals the need for additional screening measures and early diagnosis in this population. Our overall impression is that, if measures are not taken to improve screening, and curb smoking in this population and if the current conditions prevail, the rates of colon, lung, and breast cancer in the SA population will approximate those of California NHWs.

We have presented a general picture of cancer in the SA population in this paper. It is beyond the scope of this paper to discuss subtypes of each cancer. Hence we conclude that more studies are needed on this issue and subtype analysis of cancer sites needs to be conducted.

## List of Abbreviations used

**SA: **south Asian

**NHW: **non-Hispanic White

**API: **Asian/Pacific Islanders

**APC: **Annual percentage change

**CCR: **California Cancer Registry

## Authors' contributions

RVJ conceptualized and designed the study, as well as carried out the data analysis and prepared the manuscript. PKM was responsible for the study design, acquisition of funding and data, as well as interpretation of the data. APP helped prepare the manuscript and gave technical advice.
